# Blocking autophagy enhances the pro-apoptotic effect of bufalin on human gastric cancer cells through endoplasmic reticulum stress

**DOI:** 10.1242/bio.026344

**Published:** 2017-08-24

**Authors:** Hongyan Zhao, Qinghua Li, Jie Pang, Huilin Jin, Hongwei Li, Xiaoying Yang

**Affiliations:** 1 Department of Gastroenterology, the Fourth Affiliated Hospital of Harbin Medical University, Harbin 150001, China; 2 Department of Gastroenterology, the Fourth Hospital of Harbin, Harbin 150026, China; 3 Department of Hepatology and Pancreatology, Shanghai East Hospital, Tongji University, Shanghai 200120, China; 4 Pharmacy, the Fifth Hospital of Harbin, Harbin 150000, China; 5 Department of Gastroenterology, the Fourth Hospital of Harbin, Harbin 150026, China

**Keywords:** Bufalin, Endoplasmic reticulum stress, Autophagy, Gastric cancer

## Abstract

Bufalin has been used to treat cancer for several years. However, the molecular mechanisms for its anti-tumor function are not fully understood. This work aimed to investigate the effect of bufalin on the proliferation and apoptosis of human gastric cancer (HGC) cells and the roles of endoplasmic reticulum (ER) stress and autophagy in bufalin-induced apoptosis. HGC cell lines, SGC7901 and BGC823, were treated with different concentrations of bufalin or 80 nmol/l bufalin for 1, 2, 3 and 4 days. Cell counting kit-8 (CCK-8) assay and direct cell counting method were used to detect proliferation. Cell cycle arrest and apoptosis was detected using flow cytometry. Protein levels of caspase-3, -8, Bax/Bcl-2, Beclin-1, LC3, inositol-requiring enzyme 1 (IRE1) and C/EBP homologous protein (CHOP) were determined using western blotting. Autophagy was blocked using 3-methyladenine (3MA) or Atg5 siRNA to evaluate the effect of autophagy on bufalin-induced apoptosis. The IRE1 and CHOP were knocked down using specific siRNA to determine the pathway involved in bufalin-induced autophagy. It was found that bufalin significantly suppressed proliferation of SGC7901 and BGC823 cells and induced apoptosis in a time- and dose-dependent manner. The mechanism responsible for bufalin-induced apoptosis was the formation of ER stress via the IRE1-JNK pathway. Moreover, autophagy was activated during ER stress, and blocking autophagy significantly exacerbated bufalin-induced apoptosis.

## Abstract (precis)

**Summary:** Bufalin suppressed human gastric cancer cells and induced apoptosis. The mechanism was related to ER stress formation via the IRE1-JNK pathway. Blocking autophagy exacerbated bufalin-induced apoptosis.

## INTRODUCTION

Gastric cancer is one of the most common digestive malignant tumors in the world. China, Japan, and South Korea, as well as some western countries, are the high-incidence areas of gastric cancer. According to statistics, in China there are 400,000 new cases of gastric cancer every year accounting for 42% of the world's total number of cases (Yang, 2006). Although the five-year survival rate of patients with early gastric cancer is greater than 90% after surgery, the prognosis of patients with advanced gastric cancer is poor, and the five-year survival rate is around 5-20% (Bang et al., 2010). However, due to lack of specific performance, the early gastric cancer is difficult to detect. Approximately two-thirds of patients are found to have advanced gastric cancer, and can only be treated with chemotherapy and/or radiotherapy (Pozzo and Barone, 2008). Although numerous chemotherapeutic agents, such as epirubicin, fluorouracil, docetaxel and cisplatin have been used in clinical practice and made some progress in the improvement of survival rates of patients with advanced gastric cancer, drug resistance is still a common phenomenon, and it remains a great challenge to explore more efficacious drugs and therapies (Jemal et al., 2015; Martel et al., 2013).

Bufalin is a major medicinal ingredient extracted from the Chinese traditional medicine Chan Su. Accumulating studies have demonstrated that bufalin could inhibit proliferation and induce apoptosis of various tumor cell lines, including bladder cancer cells, uterine choriocarcinoma cells, breast cancer cells and liver cancer cells, and no obvious side effects have been observed (Takai et al., 2012; Li et al., 2009; Jiang et al., 2010). However, the mechanisms responsible for its anti-tumor efficacy are probably multifactorial and not fully understood. Dan Li et al. demonstrated bufalin at a high concentration (80 nmol/l) could induce human gastric cancer cell line (MGC803) apoptosis through increasing the Bax/Bcl-2 ratio, activating caspase-3 and inhibiting the PI3K/Akt pathway (Li et al., 2009). Bufalin has also been found to cause apoptosis in prostate cancer cells via a sequence of apoptotic modulators, including Bax, cytochrome c, and caspases, and the upstream mediators might be p53 and Fas (Yu et al., 2008). Chuan-Ming Xie et al. found bufalin induced human colon cancer cells (SW620) apoptosis by activation of autophagy through reactive oxygen species generation and JNK activation, which was evidenced by the accumulation of LC3-II, and increased levels of ATG5 and Beclin-1 (Xie et al., 2011).

At this time, there are very few data concerning the role of endoplasmic reticulum (ER) stress and autophagy in the progression of bufalin-induced apoptosis in human gastric cancer (HGC) lines. In this work, the effect of bufalin on the proliferation, apoptosis, and activation of ER stress and autophagy of two HGC lines, BGC823 and SGC7901, were determined. In addition, the interaction between ER stress and autophagy, and the influence of autophagy on bufalin-induced apoptosis were also investigated.

## RESULTS

### Bufalin suppressed proliferation of HGC cells and induced their cell cycle arrest

**Fig. 1.**
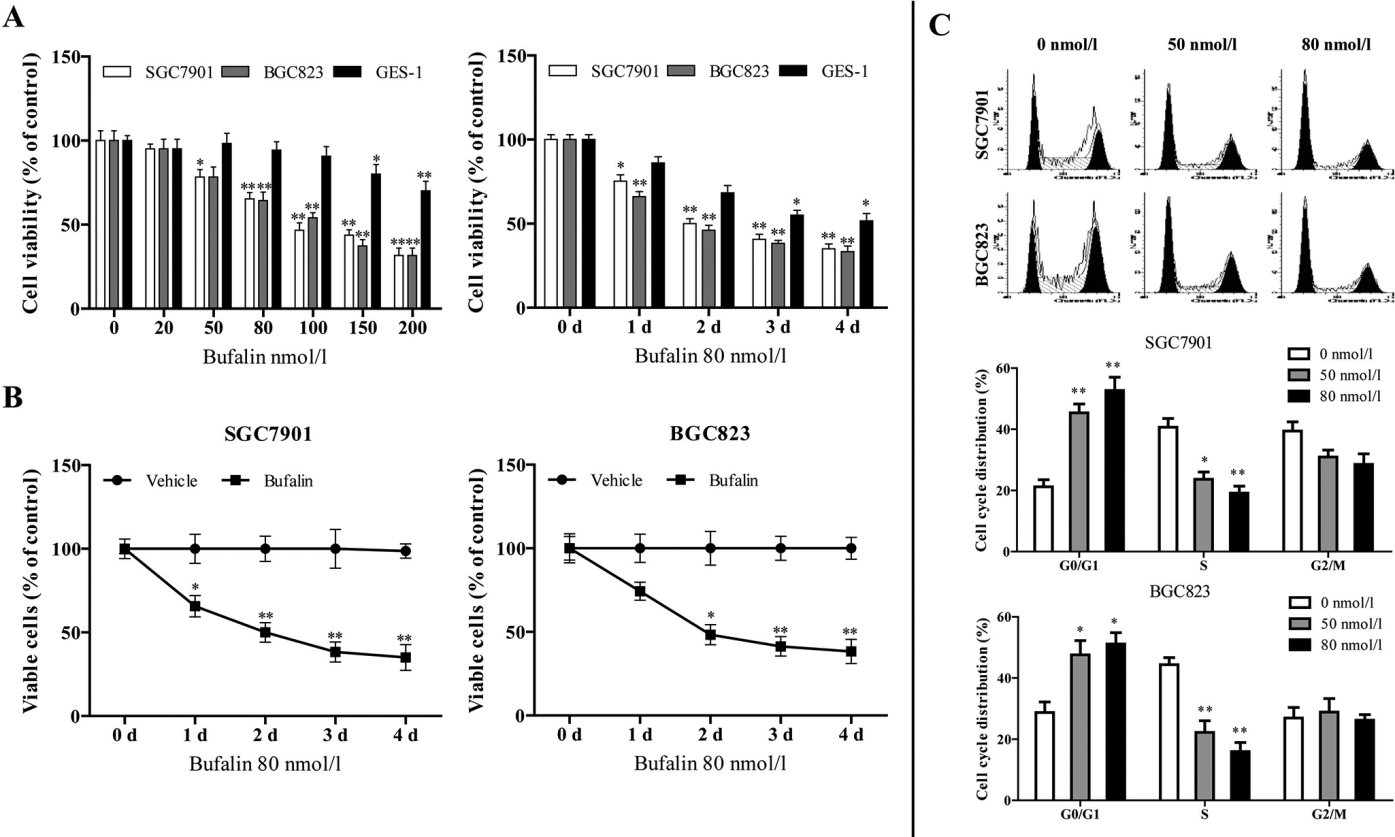
**Bufalin suppressed proliferation of HGC cells and induced their cell cycle arrest.** (A) HGC cells and a normal gastric mucous epithelium cell, GES-1, were treated with 20, 50, 80, 100, 150 and 200 nmol/l of bufalin or vehicle as control for 48 h, or 80 nmol/l of bufalin for 0, 1, 2, 3 and 4 days. Cell viability was determined using a CCK-8 assay. (B) Trypan Blue assays were performed to determine the anti-proliferative effect of bufalin on SGC7901 and BGC823 cells. (C) HGC cells were treated with 50 and 80 nmol/l of bufalin or vehicle as control for 48 h. The cell cycle distribution was determined using flow cytometric analysis and cell cycle distribution was quantified. *P<0.05 versus control treatment, **P<0.01 versus control treatment, Student's *t*-test. Data are presented as mean±s.e.

SGC7901 and BGC823 cells were treated with a series of different concentrations of bufalin (0, 20, 50, 80, 100, 150 and 200 nmol/l) for 48 h or 80 nmol/l of bufalin for the indicated durations (0, 1, 2, 3 and 4 days), and the cell viability was measured by cell counting kit-8 (CCK-8) assay. Bufalin elicited a decrease in cell viability in a dose- and time-dependent manner in HGC cells (Fig. 1A), whereas showed little influence on normal gastric mucous epithelium cell line, GES-1, until 200 nmol/l (Fig. 1A). Trypan Blue assay showed that bufalin significantly inhibited cell proliferation both in SGC-7901 and BGC-823 cell lines compared with vehicle (Fig. 1B). Flow cytometric analysis was performed to examine the effect of bufalin on proliferation of HGC cells by altering cell-cycle progression. The results demonstrated that bufalin exposure (50 or 80 nmol/l bufalin for 48 h) remarkably induced the increase of the percentage of cells in G0/G1 phase in both SGC7901 and BGC823 cells (Fig. 1C).

### Bufalin induced ER stress-mediated apoptosis in HGC cells via the IRE1-JNK pathway

**Fig. 2.**
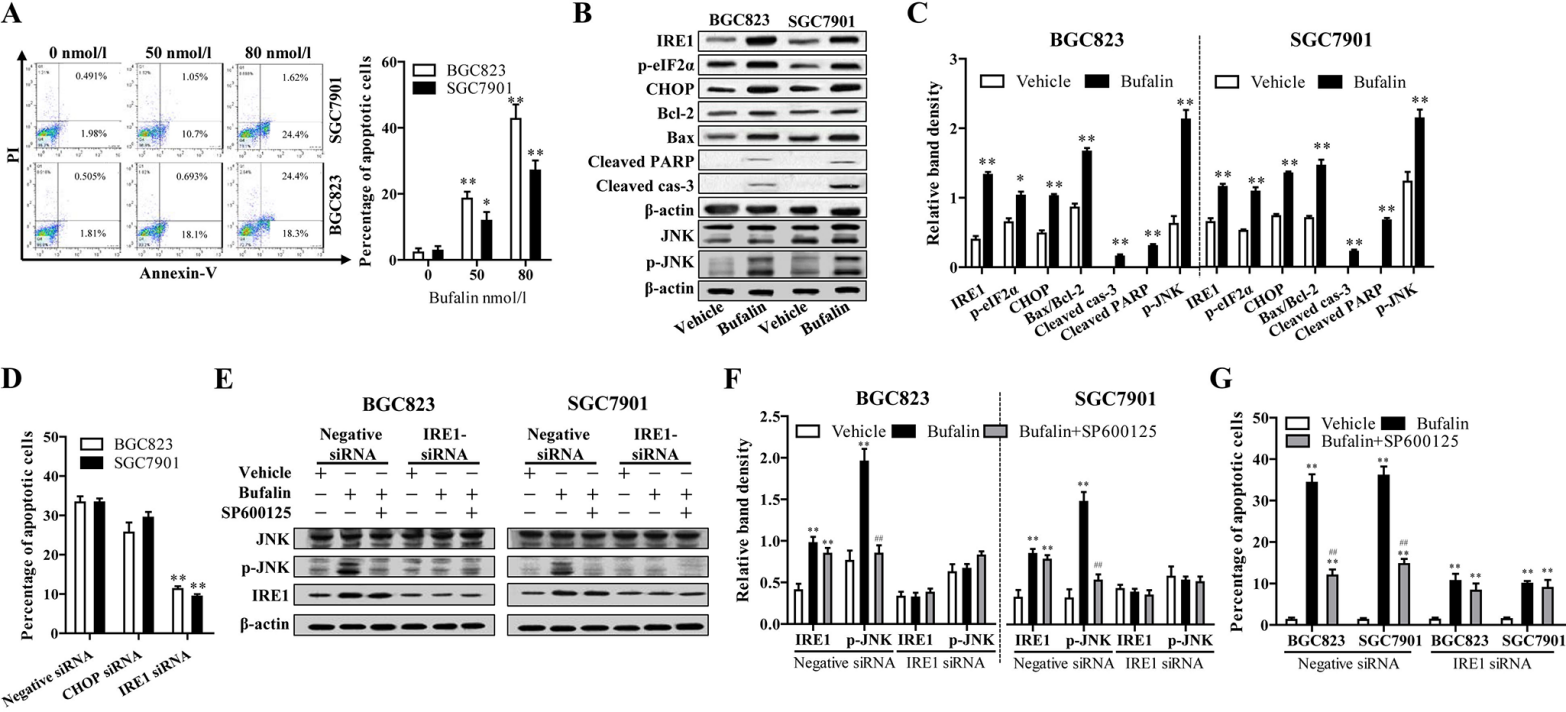
**Bufalin induced ER stress-mediated apoptosis in HGC cells via the IRE1-JNK pathway.** (A) HGC cells were treated with 50 and 80 nmol/l of bufalin or vehicle as control for 48 h and apoptosis was measured using flow cytometric analysis. (B) HGC cells were incubated with 80 nmol/l bufalin for 48 h. The expression of ER stress and apoptosis-related proteins were detected by western blot analysis. (C) The density of each protein band was measured and normalized to that of β-actin. (D) HGC cells were transfected negative siRNA and specific siRNAs against IRE1 and CHOP and exposed to 80 nmol/l bufalin. Apoptosis was measured using flow cytometric analysis after 48 h. (E,F) Specific siRNA was used to knock down the expression of IRE1 with negative siRNA as control and SP600125 (10 μmol/l) was used to block the JNK pathway with vehicle (0.1% DMSO) as control in HGC cells. Cells were exposed to 80 nmol/l bufalin for 48 h, and phosphorylation of JNK and IRE1 expression were determined by western blot analysis. (G) Apoptosis was measured using flow cytometric analysis. *P<0.05 versus control treatment, **P<0.01 versus control treatment, ^#^*P*<0.05 versus bufalin treatment, ^##^*P*<0.01 versus bufalin treatment, ANOVA post hoc test. Data are presented as mean±s.e.

To investigate the effect of bufalin on apoptosis, the apoptotic rate of SGC7901 and BGC823 cells exposed to bufalin was examined using flow cytometry. Compared with vehicle, 50 or 80 nmol/l bufalin markedly promoted apoptosis of cancer cells (Fig. 2A). To elucidate the mechanism by which bufalin induced apoptosis in HGC cells, the expression of apoptosis-related proteins was determined using western blotting assay. Our data suggested that the protein levels of cleaved caspase-3 and cleaved PARP, and the ratio of Bax/Bcl-2, were significantly increased after bufalin treatment (Fig. 2B,C), indicating that caspase and mitochondrial-mediated apoptotic pathway were involved in bufalin-induced apoptosis of gastric cancer cells. Moreover, the expression of target genes of the ER stress pathway, including inositol-requiring enzyme 1 (IRE1), C/EBP homologous protein (CHOP) and phospho-eIF2a, was significantly increased after 48 h of exposure to 80 nmol/l bufalin (Fig. 2B,C), indicating that bufalin induced ER stress in gastric cancer cells.

To determine the role of ER stress in bufalin-induced apoptosis, and which pathway was involved in the pro-apoptotic effect, CHOP or IRE1 expression in SGC7901 and BGC823 cells was reduced using siRNAs targeting CHOP or IRE1, respectively. As shown in Fig. 2D, IRE1 knockdown greatly abolished the apoptosis-inducing effect of bufalin in HGC cells, however CHOP knockdown did not. The results suggested IRE1 pathway was involved in bufalin-induced apoptosis in HGC cells.

It has been demonstrated that JNK activation by the kinase domain of IRE1 contributed to the ER stress-induced apoptosis in tubular epithelial cells (Kato et al., 2012). In parallel to the marked increase of IRE1 expression in response to bufalin, immunoblotting demonstrated that the expression of phosphorylated JNK protein also increased after bufalin treatment (Fig. 2B,C). To address whether JNK was activated by IRE1 and subsequently led to HGC cell apoptosis in response to bufalin, the cells transfected with IRE1-siRNA or negative siRNA were pretreated with the specific JNK inhibitor SP600125 (S5567, Sigma), and apoptosis was determined after bufalin exposure. As expected, SP600125 significantly attenuated bufalin-induced JNK activation in cells transfected with negative control siRNA, whereas it showed little influence on increased IRE1 expression (Fig. 2E,F). In cells transfected with IRE1-siRNA, exposed bufalin did not induce JNK activation (Fig. 2E,F). The results of apoptosis determination showed JNK inhibition using SP600125 significantly reduced bufalin-induced apoptosis in normal control HGC cells, however could not decrease the proportion of apoptotic cells when IRE1 was knocked down (Fig. 2G). In conclusion, IRE1 upregulation induced JNK activation and played a vital role in bufalin-induced HGC cell apoptosis.

### Bufalin activated protective autophagy in HGC cells

**Fig. 3.**
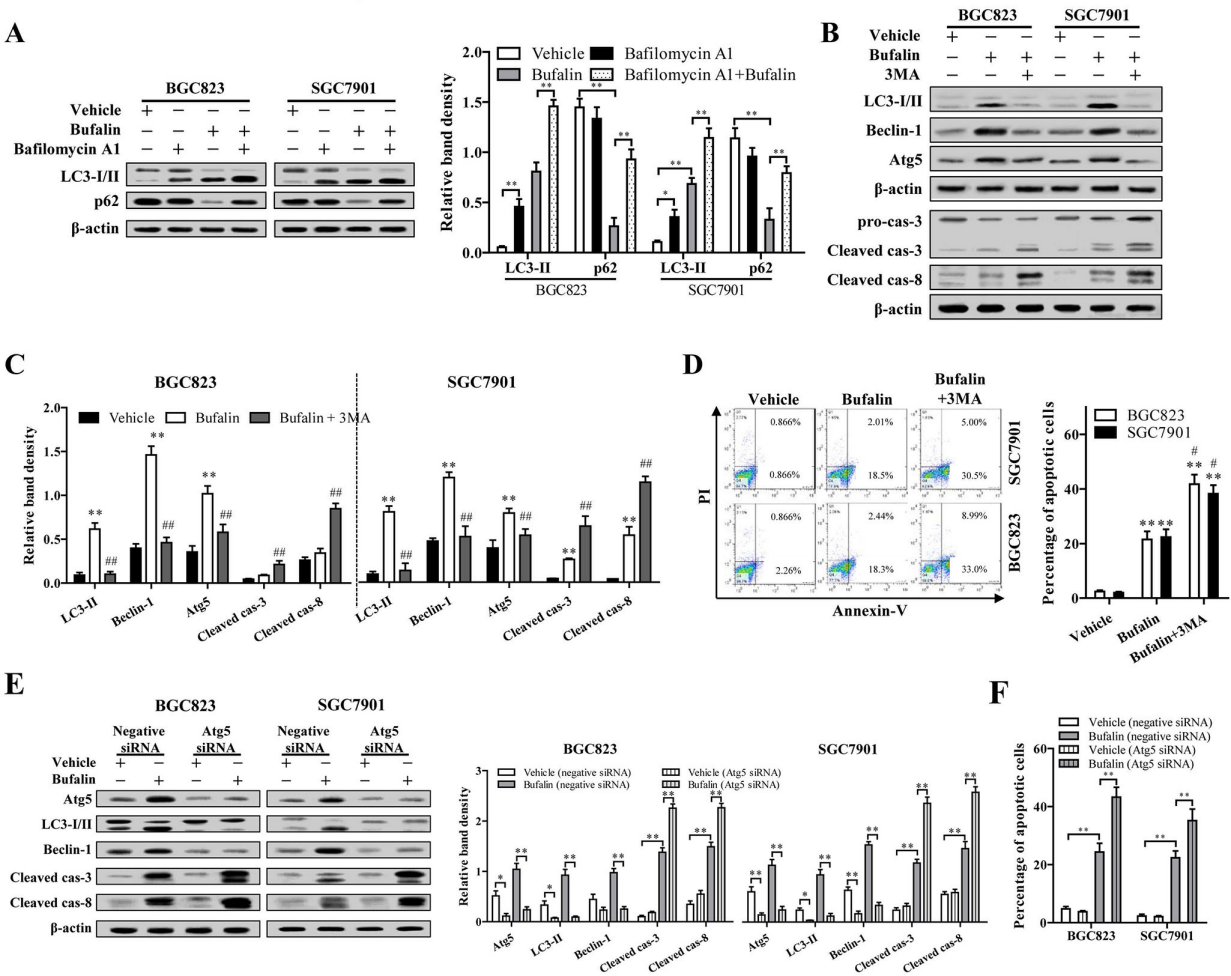
**Bufalin activated protective autophagy in HGC cells.** (A) HGC cells were treated with 80 nmol/l bufalin and/or 100 nmol/l bafilomycin A1 for 48 h, the expression of LC3-II and p62 was determined. (B) HGC cells were pretreated with 10 mM 3MA for 6 h, and then exposed to 80 nmol/l bufalin or vehicle for 48 h. The expression of LC3-I, LC3-II, Beclin-1, caspase-3, -8 and Atg5 were detected by western blot analysis. (C) The density of each protein band was measured and normalized to that of β-actin. (D) The cell apoptosis was measured using flow cytometric and the percentage of annexin V-positive cells was quantified. (E,F) Specific siRNAs against Atg5 was transfected in HGC cells. After exposed to 80 nmol/l bufalin for 48 h, the protein levels of Atg5, Beclin-1, LC3-II, cleaved caspase-3, -8 and apoptosis were determined. *P<0.05 versus vehicle, **P<0.01 versus vehicle, ^#^*P*<0.05 versus bufalin, ^##^*P*<0.01 versus bufalin, ANOVA post hoc test. Data are presented as mean±s.e.

Accumulating studies have demonstrated that protective autophagy can be activated by ER stress, which may contribute to chemotherapy resistance of lung epithelial cells, cervical cancer cells and digestive tract malignancies (Oh and Lim, 2009; Xu et al., 2012; Sui et al., 2013; Verfaillie et al., 2010). Since bufalin was found to induce ER stress in SGC7901 and BGC823 cells, the effects of bufalin on the autophagy progression in these cell lines were assessed through determination of the protein expression of LC3-I and p62. Compared to cells treated with vehicle, the levels of LC3-I and p62 significantly decreased in HGC cells treated with bufalin, whereas LC3-II increased (Fig. 3A), indicating bufalin treatment induced LC3 conversion and p62 degradation in HGC cells. Inhibition of lysosomal degradation in HGC cells exposed to bufalin using bafilomycin A1 further increased LC3-II level, and restored p62 amount to almost normal level (Fig. 3A). Taken together, bufalin induced autophagic flux increase in HGC cells.

To investigate the effect of bufalin-induced autophagy on the survival of SGC7901 and BGC823 cells, the cells were pretreated with 10 mM 3-methyladenine (3MA; M9281, Sigma) for 6 h or transfected with Atg5 siRNA to block autophagy. Inactivation of autophagy significantly exacerbated bufalin-induced apoptosis, evidenced by the increased proportion of apoptotic cells and cleaved caspase-3, and -8 (Fig. 3B-F). These data indicated that bufalin-induced autophagy is conducive to the survival of gastric cancer cells.

### The IRE1 signaling pathway is required for the activation of autophagy induced by ER stress

**Fig. 4.**
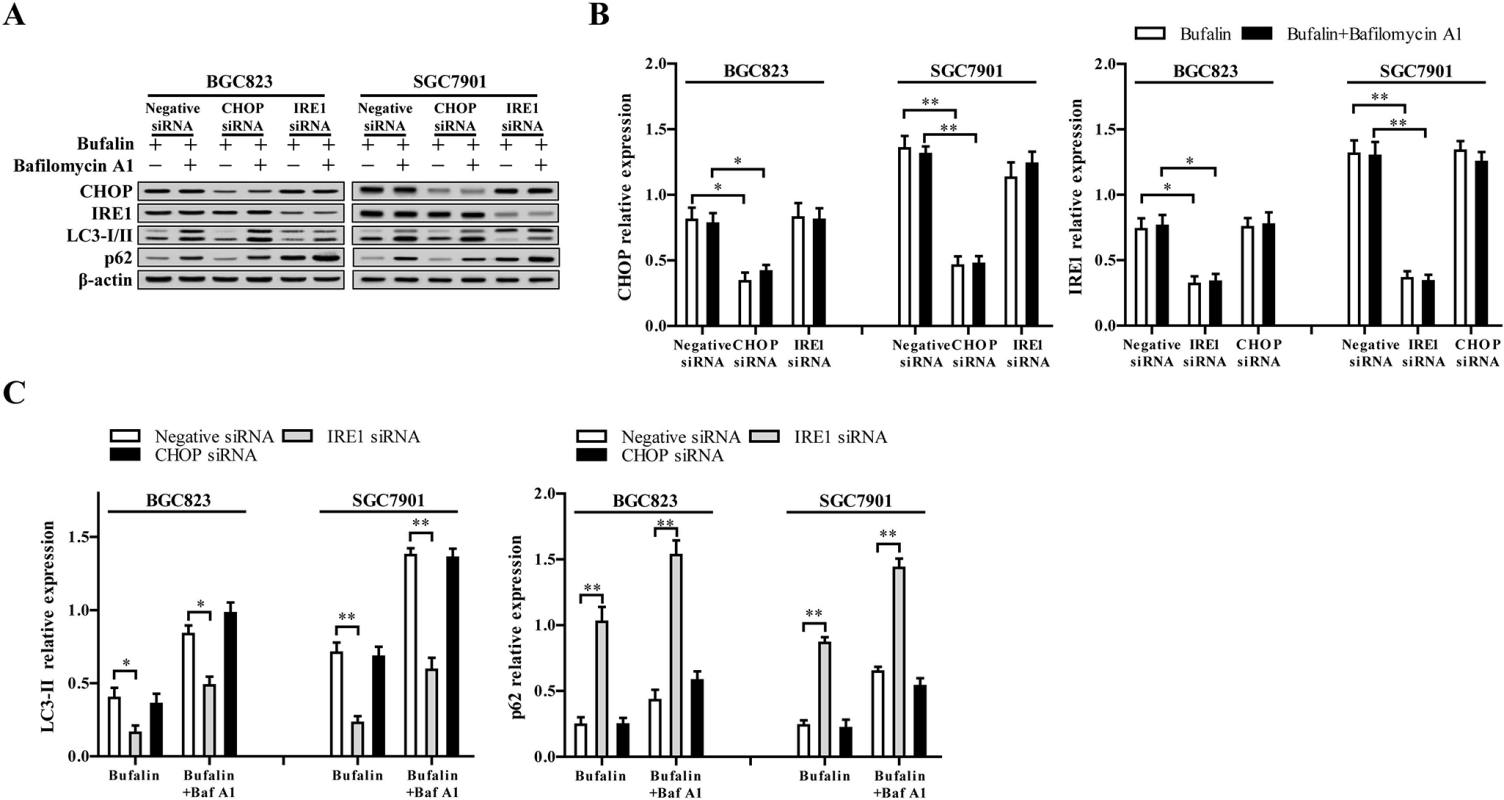
**IRE1 signaling pathway is required for the activation of autophagy induced by ER stress.** (A) Negative siRNA and specific siRNAs against IRE1 and CHOP were transfected in SGC7901 and BGC823 cell lines to knock down the expression IRE1 and CHOP. (B) After cells were exposed to 80 nmol/l bufalin for 48 h in the presence or absence of 100 nmol/l bafilomycin A1 (Baf A1), the protein levels of CHOP, IRE1, LC3-I/II, and p62 were validated. (B,C) The band density in A was measured and compared to that of β-actin to calculate relative band density. *P<0.05 versus negative siRNA, **P<0.01 versus negative siRNA, ANOVA post hoc test. Data are presented as mean±s.e.

Recently, there is evidence that when the ER stress exceeds to a certain extent, the activated unfolded protein response (UPR) triggers autophagy (Høyer-Hansen and Jäättelä, 2007). At present, the protein kinase R-like ER kinase (PERK) and IRE1 signal transduction pathway of UPR are implicated in the activation of autophagy. As mentioned above, the PERK and IRE1 are activated in SGC7901 and BGC823 cells in response to ER stress after treatment with bufalin. To determine the exact pathway involved in autophagy activation, the CHOP and IRE1 were knocked-down using siRNA, and autophagy activation was determined. CHOP and IRE1 expression were significantly decreased by CHOP siRNA and IRE1 siRNA, respectively, whether bafilomycin A1 existed or not (Fig. 4A,B). IRE1 knockdown suppressed bufalin-induced LC3 conversion and LC3-II further accumulation when autophagy was inhibited by bafilomycin A1, whereas CHOP knockdown showed little influence on the autophagy progress (Fig. 4A,C). In line with this, IRE1 knockdown also suppressed p62 degradation induced by bufalin (Fig. 4A,C). However, no significant difference was observed between CHOP knockdown cells and control cells in p62 protein levels (Fig. 4). Our data suggest that IRE1 plays a vital role in ER stress-induced autophagy following bufalin treatment.

## DISCUSSION

Bufalin is a kind of water-soluble preparation produced by the skin and parotid venom glands of the *Bufo gargarizans* Cantor, and is widely used in China to treat various cancers. Accumulating clinical and experimental research has confirmed bufalin can inhibit various human tumor cell proliferation, induce differentiation, promote apoptosis and suppress angiogenesis (Yin et al., 2012; Takai et al., 2012). However, the mechanisms responsible are poorly understood. Therefore, the elucidation of its mechanism of anti-tumor activity has great significance not only for the improvement of the accuracy of clinical medication of bufalin but also in the development of new anti-tumor drugs.

Accumulating evidence suggests bufalin could inhibit the proliferation of multiple tumor cell types, including human colon cancer cells (colon, 26-L5), leukemia cells (K562, U937, ML1 and HL-60), hepatocellular carcinoma cells (SMMC-7721 and Bel-7402), prostate cancer cells (LNCaP, DU 145 and PC3), endometrial carcinoma cells (HHUA and HEC-1), and ovarian cancer cells (SK-OV3 and omc-3) (Xian et al., 2007; Debatin and Krammer, 2004; Caldwell and Park, 2009). In this work, the proliferation of SGC7901 and BGC823 was remarkably restrained by bufalin treatment in a time- and concentration-dependent manner. Flow cytometry showed that bufalin induced HGC cell cycle arrest in G0/G1 phase. In accordance with our results, N. Takai et al. also documented that endometrial and ovarian cancer cell lines (Ishikawa, HHUA, HEC-1B, SK-OV-3 and OMC-3) were sensitive to the growth-inhibitory effect of bufalin and their exposure to bufalin increased the proportion in the G0/G1 phases of the cell cycle, which might be related to the up-regulation of p21^WAF1^ and decreased expression of cyclin A and cyclin D3 (Takai et al., 2008).

In addition to inducing proliferation inhibition, our data showed bufalin treatment also promoted HGC cell apoptosis. The apoptosis-induced effects of bufalin have been well documented in a wide spectrum of cancer cells, including lung cancer cells (A549) (Zhitu and Hongzhi, 2012), breast cancer cells (Dong et al., 2011), prostate cancer, hepatocellular carcinoma and leukemia (Yu et al., 2008; Qi et al., 2011; Yin et al., 2011; Hashimoto et al., 1997). It is also well documented that bufalin could induce apoptosis in gastric cancer. Anticancer drugs induce tumor cell apoptosis through different apoptotic pathways, such as mitochondrial pathway, death receptor-mediated signaling pathway, and ER stress-mediated pathway (Von and Vollmar, 2010). Bufalin at concentrations of 0.01 and 0.1 μmol/l could induce gastric cancer cell (MGC803) death characterized by DNA content changes to apoptotic phenotypes and chromosome DNA fragmentation (Chen, 2000). Dan Li et al. demonstrated that bufalin induced MGC803 cell apoptosis through increasing the Bax/Bcl-2 ratio, activation of caspase-3 and inhibition of phosphatidylinositol 3-kinase (PI3K)/Akt signaling pathway; and a combination of bufalin and LY294002, a PI3K-specific inhibitor, enhanced apoptosis (Li et al., 2009). Here, we found bufalin induced HGC cells apoptosis through ER stress mediated activation of the IRE1-JNK pathway.

ER dysfunction leads to the unfolded protein response, which can eventually trigger cell death if the dysfunction is extensive or prolonged (Xu et al., 2005). Existing evidence has indicated bufalin could induce ER stress in a variety of tumor cells, including glioma cells (Shen et al., 2014), hepatocellular carcinoma cells (Hu et al., 2014) and osteoblastoma cells (Zhu et al., 2014). Three molecular sensors are associated with UPR pathway, IRE1, activating transcription factor 6 (ATF6) and PERK. Activated PERK could result in CHOP activation through eukaryotic initiation factor 2 (eIF2) and activating transcription factor 4 (ATF4). In this work, IRE1, p-eIF2α and CHOP were all significantly increased in HGC cells after exposure to bufalin, indicating both IRE1 and PERK were involved in bufalin-induced HGC cells apoptosis. However, only IRE1 silence reduced bufalin-induced apoptosis, suggesting IRE1 pathway plays a vital role in the induction of apoptosis under conditions of ER stress. Similar to our study, Fengli Hu et al. also found hepatocellular carcinoma cells with silenced IRE1 expression had a higher viability after treatment with bufalin, whereas cells with silenced CHOP expression did not (Hu et al., 2014). The unexpected results may be due to the different functions of the three pathways in ER stress. Numerous studies have identified that the decision between pro-survival and pro-death outcomes in the ER stress pathway is determined by the activity of IRE1 (Chen and Brandizzi, 2013). Bufalin-induced increase in JNK phosphorylation could be reversed by siRNA targeting IRE1, which suggested JNK was downstream of IRE1. In addition, JNK inhibition using SP600125 or siRNA targeting IRE1 partly abolished bufalin-induced HGC cell apoptosis. In conclusion, the results indicated that IRE1 upregulation-induced JNK activation plays a vital role in bufalin-induced HGC cell apoptosis.

Autophagy is an important metabolic process that allows degradation and reuse of intracellular biological macromolecules and damaged organelles under certain stress conditions (Mizushima, 2007). Autophagy has a dual nature in the regulation of cell death: mild autophagy protects cells against harmful conditions and promotes cell survival; serious or quick autophagy induces programmed cell death, known as autophagic cell death (Codogno, 2005). Various cellular stresses such as ROS production, hypoxia, misfolded protein accumulation, irradiation and ER stress stimulation can induce autophagy. Moreover, autophagy can also be induced by multiple cytotoxic chemotherapeutic reagents (Tsai et al., 2012). Recent studies suggest that when the ER stress exceeded to a certain extent, the activated UPR induces autophagy. It has been discovered that PERK and IRE1 pathways are involved in the activation of autophagy. Kouroku et al. found that abnormal expression and accumulation of polyglutamine Q72 in mouse embryonic carcinoma cells and embryonic fibroblasts led to ER stress and autophagy, and knockdown of PERK-eIF2α could significantly suppress the upregulation of autophagy-related protein Atg12 and LC3-II (Kouroku et al., 2007). Some other researchers demonstrated that ER stress mediated the occurrence of autophagy through the IRE1 pathway rather than the PERK pathway, and JNK inhibitors could suppress the expression of LC3, suggesting IRE1-TRAF2-JNK pathway plays a role in the induction of autophagy by ER stress (Liu et al., 2010; Ogata et al., 2007; Tabas and Ron, 2011). Accumulating evidence also suggest that protective autophagy can be activated by ER stress, which may contribute to chemotherapy resistance in lung epithelial cells and cervical cancer cells, as well as in the digestive tract malignancies (Lim et al., 2010; Yang et al., 2010; Xu et al., 2012).

In present study, we found autophagy was induced by bufalin in SGC7901 and BGC823 cells, evidenced by the increased conversion of LC3-I to LC3-II and the degradation of p62. In addition, we found IRE1 knockdown significantly restrained LC3-II accumulation and p62 degradation induced by bufalin; whereas, CHOP knockdown showed no significant influence on autophagy progress, suggesting the IRE1 pathway was involved in ER stress-induced autophagy. To determine whether the autophagy promoted cell survival or cell death in HGC cells, autophagy was blocked with 3MA pretreatment or Atg5 siRNA. Autophagy inhibition significantly promoted bufalin-induced apoptosis, suggesting that bufalin induced protective autophagy in HGC cells. In accordance with our reports, Shuying Shen et al. reported that blockage of autophagy increased expression of ER stress-associated proteins and the ratio of apoptosis, suggesting autophagy played a cytoprotective role in bufalin-induced ER stress and cell death (Shen et al., 2014). By contrast, Chuan-Ming Xie et al. demonstrated bufalin induces autophagy-mediated cell death in HT-29 and Caco-2 human colon cancer cells through reactive oxygen species generation and JNK activation (Xie et al., 2011). This may be explained by the difference in tumor cell types and concentration of bufalin.

In conclusion, the present study demonstrated that bufalin suppressed the proliferation of human gastric cancer cell lines, SGC7901 and BGC823 in a concentration- and time-dependent manner, and promoted ER stress-mediated apoptosis. In addition, bufalin-induced ER stress led to the occurrence of autophagy through IRE1 pathway and JNK activation. Blockage of autophagy accelerated bufalin-induced apoptosis in HGC cells.

## MATERIALS AND METHODS

### Cell culture

HGC cell lines BGC823, SGC7901 and one normal gastric mucous epithelium cell (GES-1) were obtained from the Type Culture Collection Cell Bank (Chinese Academy of Sciences Committee, Shanghai, China) and were routinely cultured in RPMI 1640 (GIBCO) supplemented with 10% fetal bovine serum (FBS; EU GIBCO), 100 U/ml penicillin and 10 μg/ml streptomycin in a humidified atmosphere of 5% CO_2_ at 37°C.

### Cell proliferation assays

The cell viability was determined using the CCK-8 kit based on the manufacturer's instructions. BGC823, SGC7901 and GES-1 were plated at a density of 3×10^3^ cells per well in 96-well microtiter plates and cultured overnight at 37°C in a humidified incubator containing 5% CO_2_. After treatment with 20, 50, 80, 100, 150 and 200 nmol/l of bufalin for 48 h, or 80 nmol/l of bufalin for 0, 1, 2, 3 and 4 days, the culture medium was replaced with 100 μl of fresh medium followed by the addition of 10 μl of CCK-8 solution. The cells were further incubated for 2 h before the optical density at 450 nm was recorded. The effect of bufalin on proliferation was assessed by Trypan Blue exclusion assay (Beyotime, Haimen, China). Trypan Blue staining was evaluated under the microscope and the number of viable cells (unstained blue) was counted using a hemocytometer.

### Detection of cell cycle

Phase distributions of the cell cycle were determined by flow cytometry. Cells were seeded at a density of 1.5×10^6^ cells per well in 6-well plates, and exposed to 0, 50 and 80 nmol/l bufalin for 48 h. Then the cells were harvested, washed twice with PBS and fixed with 75% ethanol at 4°C for 12 h. After washing twice with PBS, the cells were incubated with 20 mg/ml RNase A and 10 mg/ml PI for 30 min in the dark. Finally, the cells were analyzed with flow cytometer (Becton-Dickinson Immunocytometery Systems, San Jose, CA, USA) and the data were analyzed using Cell Quest software (Becton Dickinson, San Jose, CA, USA).

### Detection of cell apoptosis

Cell apoptosis was detected using Annexin V/PI staining and flow cytometry. Cells were seeded at a density of 1.5×10^6^ cells per well in 6-well plates. After treatment with different reagents for 48 h, the cells were collected, washed with PBS, and stained with fluorescein isothiocyanate (FITC)-Annexin V and propidium iodide (PI) by the FITC Annexin V Apoptosis Detection Kit (BD Biosciences, Shanghai, China) according to the manufacturer's recommendations. Flow cytometric analysis was performed immediately using a Beckman Coulter Epics Altra II cytometer (Beckman Coulter, CA, USA).

### RNA interference

To knock down the gene expression of IRE1, CHOP and Atg5, the sense sequences of siRNA oligonucleotides targeting the transcripts of IRE1, CHOP and Atg5 were transfected into BGC823 and SGC7901 cells using the Lipofectamine 2000 (Invitrogen). Mismatch sequences were used as a negative control. Sequences for the IRE1, CHOP and Atg5 siRNAs were described in previous literature (Shi et al., 2011). After incubation with siRNAs for 48 h, the cells were treated with bufalin for 48 h and harvested for use in cell apoptosis and western blot assays.

### Western blot analysis

The cells treated with different reagents for 48 h were collected and lysed using RIPA lysis buffer with proteinase inhibitor. The determination of total protein concentration was performed using the Protein BCA Assay Kit (Bio-Rad, USA). Then equal amounts of cell lysate mixed with 2× SDS loading buffer were separated by 12% SDS-PAGE and transferred to polyvinylidene difluoride membranes (Millipore, USA). After blocking with 10% non-fat milk in Tris-buffered saline containing 0.1% (v/v) Tween-20 (TBS-T) for 50 min, the membrane was incubated with 10% nonfat milk containing primary antibodies against IRE1 (1:1000, #3294, CST), p-eIF2α (1:1000, #9721, CST), CHOP (1:1000, #2895, CST), Bcl-2 (1:500, sc-509, Santa Cruz Biotechnology), Bax (1:500, sc-20067, Santa Cruz Biotechnology), PARP (1:500, sc-7150, Santa Cruz Biotechnology), caspase-3 (1:1000, #9665, CST), caspase-8 (1:1000, #9746, CST), JNK (1:1000, #9251, CST), LC3 (1:1000, #2775, CST), Beclin-1 (1:1000, #3738, CST), Atg5 (1:1000, #2630, CST), p62 (1:2000, ab56416, Abcam) and β-actin (1:1000, #4967, CST) for 2 h at room temperature. After washing, the membrane was incubated with appropriate secondary antibodies conjugated to horseradish peroxidase for 1 h at room temperature. The bands were visualized with ECL western blotting detection reagents. An anti-β-actin antibody was used as a protein loading control.

### Statistical analysis

All experiments were performed in triplicate and repeated at least three times. Data are expressed as mean±s.e. Statistical analysis was performed using Student's *t*-test for comparison of two groups or ANOVA post hoc test for comparison of more than two groups. A *P*-value of less than 0.05 was considered statistically significant.
